# Expression, Purification, and Characterization of Ras Protein (BmRas1) from *Bombyx mori*

**DOI:** 10.1155/2012/747539

**Published:** 2012-03-29

**Authors:** Yanping Quan, Guangqiang Liu, Wei Yu, Zuoming Nie, Jian Chen, Zhengbing Lv, Yaozhou Zhang

**Affiliations:** ^1^Institute of Biochemistry, College of Life Sciences, Zhejiang Sci-Tech University, Zhejiang Province, Hangzhou 310018, China; ^2^Zhejiang Provincial Key Laboratory of Silkworm Bioreactor and Biomedicine, Zhejiang Province, Hangzhou 310018, China

## Abstract

The Ras subfamily is the member of small G proteins superfamily involved in cellular signal transduction. Activation of Ras signaling causes cell growth, differentiation, and survival. *Bombyx mori* Ras-like protein (BmRas1) may belong to the Ras subfamily. It contained an H-N-K-Ras-like domain. The BmRas1 mRNA consisted of 1459 bp. The open reading frame contained 579 bp, encoding 192 amino acids. The protein had such secondary structures as **α**-helices, extended strand, and random coil. BmRas1 was expressed successfully in *E. coli* BL21. The recombinant protein was purified with metal-chelating affinity chromatography. The GTPase activity of purified protein was determined by FeSO_4_-(NH_4_)_2_MoO_4_ assay. The results showed that purified recombinant protein had intrinsic activity of GTPase. High titer polyclonal antibodies were generated by New Zealand rabbit immunized with purified protein. The gene expression features of BmRas1 at different stages and in different organs of the fifth instar larvae were analyzed by Western blot. The results showed that BmRas1 was expressed highly in three development stages including egg, pupae, and adult, but low expression in larva. BmRas1 was expressed in these tissues including head, malpighian tubule, genital gland, and silk gland. The purified recombinant protein would be utilized to further function studies of BmRas1.

## 1. Introduction

Ras genes were first identified as homologues of rodent sarcoma virus genes. In 1982, human DNA sequences homologous to the transforming oncogenes of the v-Harvey (H-Ras) and Kirsten (K-Ras) rat sarcoma virus were identified in DNA sequences derived from a human bladder and a human lung cancer cell line, respectively. There are three mammalian Ras proteins: H-Ras, N-Ras, and K-Ras, which consisted of 188-189 amino acid (p21 proteins), encoded by three ras genes [1]. The Ras isoforms are highly homologous [2]. Ras proteins are positioned at the inner surface of the plasma membrane where they serve as binary molecular switches to transduce extracellular ligand-mediated stimuli into the cytoplasm to control signal transduction pathways that influence cell growth, differentiation, and apoptosis [3, 4]. The Ras protein is the prototype of the Ras superfamily of small GTPases, which share a high degree of sequence similarity and a common three-dimensional structure, called the GTP-binding domain. This domain enables them to act as molecular switches cycling between two defined conformational states: an inactive guanosine-diphosphate (GDP-) bound and an active guanosine-triphosphate-(GTP-) bound state [3, 5, 6]. The guanine nucleotide exchange factors (GEFs) promote formation of the active Ras-GTP complex by inducing dissociation of bound GDP to allow association of the more abundant GTP, thus increasing the rate of intracellular exchange of GDP for GTP [5, 7–9].

Studies in *Caenorhabditis elegans*, *Drosophila*, and mammalian cells established the mode of action of Ras proteins [10–12]. Ras couples the signals of activated growth factor receptors to downstream effectors that interact with the active GTP-bound form of Ras. Ras effectors include protein kinases, lipid kinases, and GEFs, which transmit signals to cell nuclear, recruitment to the plasma membrane, and association with substrates [13–15]. Of these, the best characterized are the Raf kinases, also referred to as the mitogen-activated protein kinase (MAPK) cascade. MAPK modules include the ERK pathway, the SAPK/JNK pathway, and the p38 pathway [7, 16]. MAPK pathways are well-conserved major signaling systems involved in the transduction of extracellular signals into cellular responses in a variety of organisms The MAPK cascades activate various substrates in the cytoplasma and the nucleus of the cell, including transcription factors. These downstream targets control cellular responses (e.g, apoptosis, proliferation, and differentiation) [12, 17, 18].

The COOH-terminal regions of small GTP-binding proteins are classified into at least four groups: (1) Cys-A-A-X (A, aliphatic acid; X, any amino acid); (2) Cys-A-A-Leu/Phe; (3) Cys-X-Cys; (4) Cys-Cys [19, 20]. The Cys-A-A-X structure is furthermore subclassified into two groups: one has an additional Cys residue upstream of the Cys residue of the Cys-A-A-X structure and the other has a polybasic region. In the case of the Cys-A-A-X structure, H-Ras and K-Ras are first farnesylated at the Cys residue followed by the proteolytic removal of the A-A-X portion and the carboxyl methylation of the exposed Cys residue [21–23].

*Bombyx mori* was studied to excavate its potential economic value and to explore the molecular mechanisms of the physiological development in lepidoptera insects as a model species. The silkworm genome has 28 chromosome pairs containing 4.8 billion base pairs. The complete genome was sequenced and analyzed, 18,510 genes were estimated [24]. In our laboratory, a cDNA library of silkworm pupae was constructed and the whole cDNA sequencing had been performed. We found a gene named* Bombyx mori* ras-like protein 1 (*BmRas1*) (GenBank accession no. NM_001043508) from the cDNA library. BmRas1 contained an H-N-K-Ras-like domain. It may be involved in the regulation of cell growth. Here we described the expression, purification, and biochemical characterization of functional BmRas1 using an *E. coli* expression system. The purified recombinant protein BmRas1 was detected with GTPase activity. BmRas1 was expressed in tissue throughout four developmental stages. Subcellular localization showed BmRas1 was found on membrane, partly in cytoplasm. The further studies aimed to understand the role of BmRas1 in development and biological function of *Bombyx mori*.

## 2. Materials and Methods

### 2.1. Animals and Tissues

The *Bombyx mori* strain used in this study is the progeny of Qingsong × Baiyu. Silkworms were reared on mulberry leaves at 25°C and 60–90% relative humidity in natural light. Fifth instar larvae, pupae, moths, and nascent eggs were frozen in liquid nitrogen and stored at −80°C. Malpighian tubule, head, epidermis, fatty body, seminal glands, ovary, and silk glands were dissected from fifth instar larvae, frozen immediately in liquid nitrogen, and stored at −80°C.

### 2.2. Bioinformatics Analysis

The protein sequences of Ras homology proteins in some species were retrieved from NCBI Protein database. Amino acid sequence of BmRas1 protein was compared with those of some members of the Ras family, which included* BmRas2 *(AB206960), *BmRas3* (AB170011), *Aedes aegypti *(EAT46745, EAT38763, EAT35784), *Anopheles gambiae *(XP_307965), *Tribolium castaneum* (XP_975587), *Xenopus laevis* (AAA49944), *Mus musculus *(NP_032310, NP_056461), *Homo sapiens *(NP_004976, NP_056461, NP_036382), *Drosophila melanogaster *(NP_476857, NP523917, NP476699),* Caenorhabditis elegans* (NP_502213), *Apis mellifera* (XP_394288, XP_393035), and *Nasonia vitripennis* (XP_001608221). Alignments of BmRas1 and Ras homology protein sequences were performed using the Jotun Hein method in DNAStar.

### 2.3. Plasmid Construction

A cDNA encoding BmRas1 was obtained from the cDNA library of the metaphase pupae constructed by our laboratory. Based on the cDNA sequence, two primers were designed as follows: 5′-GGGAATTCATGTCTCGAGCAGGCGACAGAC-3′ and 5′-CCCTCGAGTTAAAAAAGGGTGCAATC-3′, including restriction enzyme sites for *Eco*R I and *Xho *I, respectively. The predicted open reading frame was amplified. The PCR was performed for denature at 95°C for 5 min; 35 cycles of 94°C for 30 s, 60°C for 30 s, 72°C for 1 min, followed by a 10-min extension at 72°C. The PCR products were purified using the E.Z.N.A.Cycle Pure Kit (Omega, USA). After digestion with *Eco*R I and* Xho *I, the interested fragment was ligated into the expression vector pET-28a (+) and transformed into *E. coli* TG1 competent cells. pET-BmRas1, the positive plasmid colony with the BmRas1 gene, was sequenced subsequently by ABI 3130-xl Genetic Analyzer.

### 2.4. Protein Expression and Purification

The recombinant expression plasmid, pET-BmRas1, was transformed into *E. coli* BL21 (DE3). Bacterial cultures were incubated at 37°C in LB medium containing kanamycin until an OD_600_ of 0.5 was reached. Recombinant protein expression was induced by the addition of IPTG to a final concentration of 0.1 mM. Following 4 h incubation at 37°C, bacteria were harvested by centrifugation and frozen at −20°C. Bacterial pellets were resuspended with lysis buffer (50 mM Tris-HCl, 2 mM EDTA, 100 mM NaCl, pH 8.0) and lysed by pulsed sonication. Briefly, cell suspension was sonicated with 30 short bursts of 10 sec followed by intervals of 20 sec for cooling, with an Ultrasonic Crasher Φ2 cell disruptor (Ningbo Scientz, China). Keep the suspension at all times on ice. The lysates were centrifuged at 14,000 g for 20 min at 4°C. The supernatant was collected and filtered through a 0.45 *μ*m filter (Millipore, USA). The filtrate was subjected to metal chelation column chromatography using Ni-NTA His·Bind resin (Novagen) to purify the recombinant protein, as instructed by the manufacturer. The recombinant BmRas1 proteins were separated on SDS-PAGE and verified by immunoblotting with antibodies specific for the recombinant proteins.

### 2.5. GTPase Activity Assay

GTPase activity of purified BmRas1 was assayed by the FeSO_4_-(NH_4_)_2_MoO_4_ method [25]. The purified BmRas1 protein was transferred into dialysis bag to renaturation. The refolded protein (100 *μ*g/mL) was added to the reaction buffer containing 100 mM Tris-HCl (pH 8.0), 0.4 mM DTT, 650 *μ*M GTP, 10 mM MgCl_2_ to 10 *μ*g/mL. Controls were the same reaction buffer without the BmRas1 protein or GTP. The reaction mixture was incubated at 37°C for 0, 30, 40, 50, 60, 75, 90, 105, and 120 min. 25 *μ*L aliquots of the reaction were quenched with 5 *μ*L of 20% trichloroacetic acid (TCA). Added ddH_2_O to the final volume of 50 *μ*L, and centrifuged at 12.000 g for 15 min. The supernatant was added 200 *μ*L FeSO_4_-(NH_4_)_2_MoO_4_ solution containing 0.7% FeSO_4_, 0.14% (NH_4_)_2_MoO_4_, 5 mM H_2_SO_4_. The amounts of hydrolyzed inorganic phosphate were measured at 660 nm with SpectraMax Plus384 Absorbance Microplate Reader (Molecular Devices, USA).

### 2.6. Antibody Preparation

A male New Zealand white rabbit was immunized with 1 mg of purified recombinant BmRas1 protein emulsified with Freund's complete adjuvant (Sigma). Three booster doses were given at intervals of 14 days with the half amount of antigen and Freund's incomplete adjuvant (Sigma). Blood was collected after 1 month of last immunization, and the serum was isolated. The polyclonal antibody was purified by Protein A chromatography (Sigma) following the manufacturer's instructions. The purified antibody was used for immunoblotting and immunofluorescence.

### 2.7. Subcellular Localization

BmN cells were cultured to 60–70% confluence on the confocal dish. Cells were rinsed twice with 1 mL PBS, and fixed in 3.7% formaldehyde at 25°C for 10 min. After being washed three times with PBS, cells were blocked with 3% BSA at 37°C for 2 h, and then incubated with anti-BmRas1 IgG (1 : 1000 dilutions) at 4°C for 12 h in contrast with negative serum as negative control. After washing three times in PBS with 0.05% Tween-20, cells were incubated with Cy3-labeled goat anti-rabbit antibody (1 : 1000 dilutions, Promega) and DAPI (1 : 2000 dilution, Promega) at 37°C for 2 h, following three washes in PBS with 0.05% Tween-20. Stained cells were viewed by Nikon ECLIPSE TE2000-E Confocal Microscope with image analysis software EZ-C1 3.8.

### 2.8. Tissues Localization

The distributions of BmRas1 in different tissues in silkworms were analyzed by Western blot. Eggs, the fifth instar larvae, pupae, moths, and tissues isolated from fifth instar larvae were ground to powder in liquid nitrogen. Powders were suspended in buffer (50 mM Tris pH 8.0, 0.15 M NaCl, 5 mM EDTA, 0.5% NP-40, 1 mM DTT, 5 g/L sodium deoxycholate, 100 mg/L PMSF, 5 *μ*g/mL Aprotin) and incubated for 30 min on ice. Homogenates were centrifuged at 12 000 g for 15 min at 4°C. Protein concentrations in all samples were equalized before SDS-PAGE. Protein extracts from each tissue were separated by 12% SDS-PAGE, and electrotransferred onto PVDF membranes (Millipore). After blocking with 3% nonfat milk in PBS at 4°C overnight, the membranes were probed with rabbit anti-BmRas1 antibody at room temperature for 2 hours. After washing with PBST, the membranes were incubated with HRP-conjugated goat anti-rabbit IgG (Bio-Rad Laboratories). The bands were detected by staining with diaminobenzidine method.

## 3. Results

### 3.1. Bioinformatics Analysis

**Figure 1:**
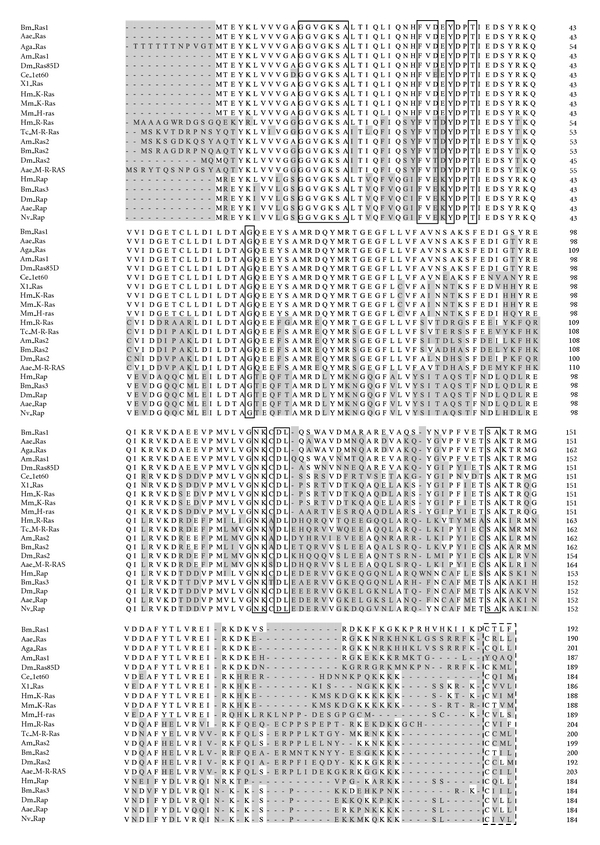
Alignment of amino acid sequences of Ras superfamily members. Bm, Aae, Aga, Am, Dm, Ce, Xl, Hm, Mm, Tc, and Nv mean *Bombyx mori*, *Aedes aegypti*, *Anopheles gambiae*, *Apis mellifera*, *Drosophila melanogaster*, *Caenorhabditis elegans*, *Xenopus laevis*, *Homo sapiens*, *Mus musculus*, *Tribolium castaneum*, and *Nasonia vitripennis*, respectively. The residues with solid light gray shade differed from BmRas1. Amino acids which are important for GTP/Mg^2+^ binding were boxed in real lines. The C-terminal CAAX motif were boxed in broken lines.

The complete mRNA of the *BmRas1* was 1459 bp with an open reading frame (ORF) of 579 bp encoding a protein of 192 amino acids. The predicted molecular weight of the protein was 21.8 kD and the theoretical isoelectric point was 6.33. A homology search using BLAST (http://blast.ncbi.nlm.nih.gov/) revealed that conserved domain of BmRas1-specific hits H-N-K-Ras-like subfamily. Amino acid sequences were aligned using the algorithm Jotun Hein Method in the software package DNAStar (Figure 1). Amino acid residues of Ras superfamily members which were important to GTP/Mg^2+^-binding site, GEF interaction site, Switch I and Switch II region were conserved in the BmRas1. The amino acids 32–40 and amino acids 60–76 domains were generally referred to as the switch I and switch II domains. GEF interaction site was within the switch II domain of Ras, as residues 62–69.

The carboxy-terminal sequences of the different Ras isoforms comprising 20–25 amino acids, termed “the hypervariable region” (HVR), were highly varied between the different Ras proteins. A C-terminal CAAX motif was in diversity, which undergoes posttranslational prenylation by cymosely farnesyl transferase to generate S-farnesyl cysteine thioester, followed by proteolytic cleavage of the AAX sequence and methyl esterification of the resulting C-terminal isoprenylated cysteine in the ER.

### 3.2. Protein Expression and Purification

**Figure 2:**
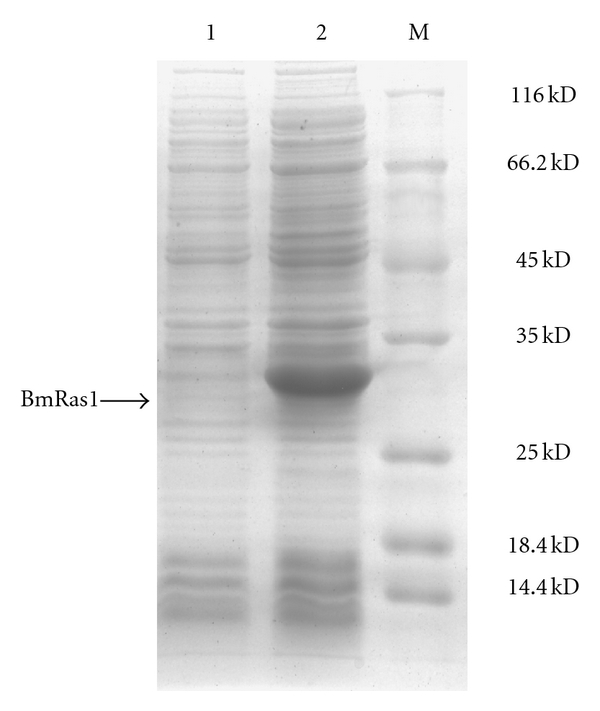
Analysis of the recombinant BmRas1 protein in SDS-PAGE. Lane 1, lysates from *E. coli* cultures transformed with pET-28a vector plasmid after IPTG induction. Lane 2, lysates from* E. coli *cells transformed with pET-28a-BmRas1 plasmid after IPTG induction. Lane M, Protein molecular weight marker.

**Figure 3:**
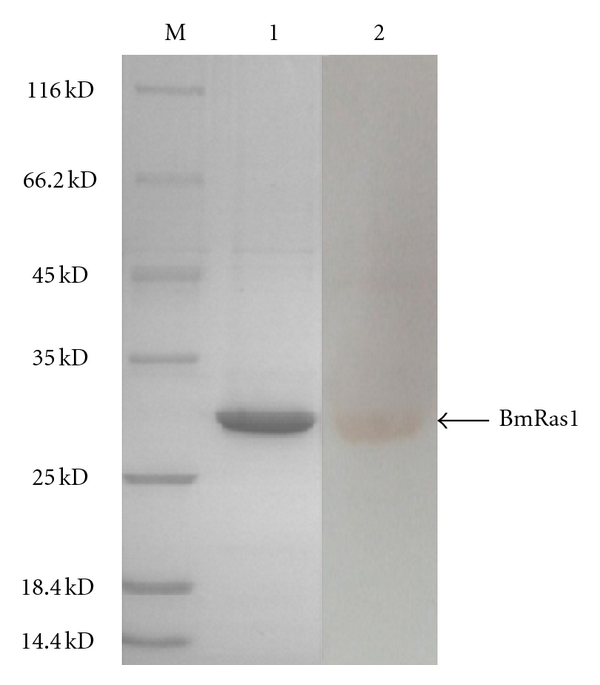
Western blot analysis of purified BmRas1 protein. Lane M. Protein molecular weight marker. Lane 1, recombinant BmRas1 was purified by nickel metal affinity resin columns and separated by SDS-PAGE. Lane 2, purified protein was analyzed by Western blot.

The ORF for BmRas1 was ligated into the expression plasmid, pET-28a, with a 6 × His tag. The fusion protein His-BmRas1 was successfully expressed in *E. coli*. Recombinant proteins were separated by 12% SDS-PAGE and analyzed by Coomassie Blue staining (Figure 2). Recombinant protein was purified using Ni-NTA His·Bind resin (Novagen) columns. Purified proteins were separated by SDS-PAGE and recognized by anti-BmRas1 antibody in Western blot (Figure 3).

### 3.3. GTPase Activity Assay

**Figure 4:**
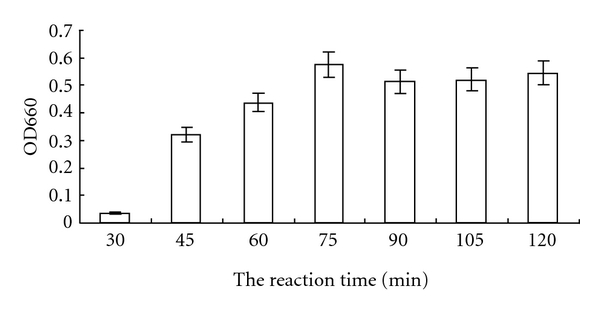
Time course of GTP hydrolysis by BmRas1. GTPase activity of purified BmRas1 was assayed by the FeSO_4_-(NH_4_)_2_MoO_4_ method to detect the amount of inorganic phosphorus. Purified proteins were incubated in the reaction mixture at 37°C for 0, 30, 45, 60, 75, 90, 105, and 120 min. At 30 min, inorganic phosphorus was detected. The amount of inorganic phosphorus was increased significantly with the time, reaching the peak at 75 min and remaining at the similar high level.

GTPase activity of purified BmRas1 was assayed by the FeSO_4_-(NH_4_)_2_MoO_4_ method to detect the amount of inorganic phosphorus. Purified proteins were incubated in the reaction mixture at 37°C for 0, 30, 45, 60, 75, 90, 105, and 120 min. At 30 min, inorganic phosphorus was detected. The amount of inorganic phosphorus was increased significantly with the time, reaching the peak at 75 min and remaining the similar high level (Figure 4). The results showed that the purified recombinant BmRas1 protein had GTP-binding activity and hydrolysis activity without accessory protein.

### 3.4. Subcellular Localization

**Figure 5:**
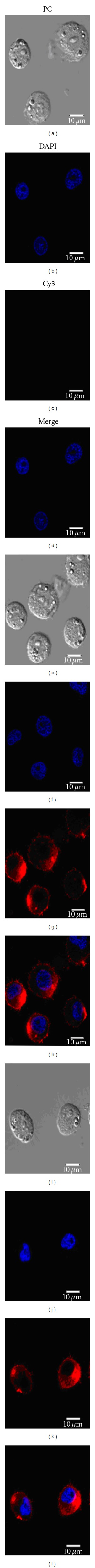
Subcellular localization of BmRas1 protein in BmN cells. Immunostaining with antibody to BmRas1 showed that BmRas1 was located on membrane, partly in cytoplasm in BmN cells. (a), (e), (i) cells in the light transmission; (b), (f), (j) nucleolus dyed by DAPI; (c), (g), (k) intracellulare BmRas1 dyed by Cy3; (d), (h), (l) merged image; (a–d) were negative control. Scar bars indicated 10 *μ*m.

Ras proteins function as signal molecule switch, anchoring in the internal leaflet of the plasma membrane, which relay signals from a number of different cell-surface receptors to the interior of the cells and induce specific cellular responses resulting in cell growth and differentiation. Immunostaining with antibody to BmRas1 showed that BmRas1 was located on membrane, partly in cytoplasm in BmN cells (Figure 5). 

### 3.5. Tissue Distribution

**Figure 6:**
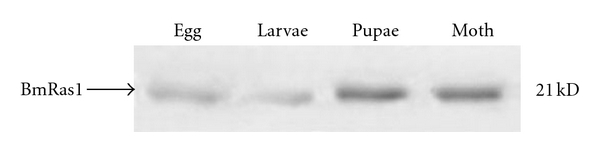
Expression of BmRas1 protein in different stages of *Bombyx mor*i. BmRas1 expressions in four developmental stages were analyzed by Western blot. BmRas1 was expressed at high level in egg, pupae and moth, but at low level in fifth instar larva.

**Figure 7:**
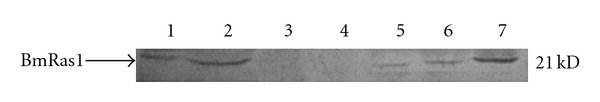
Expression of BmRas1 protein in different tissues of the fifth instar larvae of *Bombyx mori *was analyzed by Western blot. The BmRas1 protein was highly expressed in malpighian tubule, head, silk glands, and lowly expressed in seminal glands and ovary. No expression of BmRas1 was detected in epidermis and fatty body. 1: malpighian tubule; 2: head; 3: epidermis; 4: fatty body; 5: seminal glands; 6: ovary; 7: silk glands.

The gene expression levels of BmRas1 during different silkworm developmental stages and tissues distribution in fifth instar larvae were detected by Western blot. BmRas1 was expressed throughout four developmental stages (Figure 6). BmRas1 was expressed at high level in egg, pupae, and moth but at low level in fifth instar larva. BmRas1 was highly expressed in malpighian tubule, head, silk glands, and lowly expressed in seminal glands and ovary. No expression of BmRas1 was detected in epidermis and fatty body (Figure 7).

## 4. Discussion

Many different receptors at the cellular surface are expressed which allow cellular response to extracellular signals provided by the environment. Receptor activation by binding of ligand leads to a variety of biochemical events in which small GTPases are crucial. Ras proteins, a member of small GTPases family, play a key role in signal transduction, proliferation, and malignant transformation. The Ras branch of the Ras superfamily presently comprises 20 proteins, that belong to various subgroups: Ras (H-Ras, K-Ras with two alternatively spliced variants expressing the A or B fourth exon, and N-Ras), Rap (with the Rap1 A and B proteins, and Rap2 A, B and C proteins), Ral (A and B), R-Ras (comprising the R-Ras, R-Ras2/TC-21 and R-Ras3/M-Ras proteins), Rit/Rin, Rheb, Di-Ras (1 and 2), and ARHI proteins [26]. The ras genes encode 21 kDa proteins. Ras functions as a relay switch that is positioned downstream of cell surface receptor tyrosine kinases and upstream of a cytoplasmic cascade of kinases that included the mitogen-activated protein kinases (MAPKs). Activated MAPKs in turn regulated the activities of nuclear transcription factors between the cell surface and the nucleus in signaling cascade where it was defined and conserved in worms, flies, and man [2]. It is becoming increasingly evident that different members of the Ras subfamily may have different biological functions that depend not only on differences in their affinities to regulators or effectors but also in their precise subcellular localization [9]. The functions of Ras protein had been investigated in *Drosophila melanogaster* [27–31], but the study on the biological functions of Ras protein in *Bombyx mori* as representation of Lepidoptera insect was largely unknown [32, 33].

We conducted the research on the gene named *Bombyx mori* Ras protein (BmRas1) (GenBank accession no: NM_001043508) from the cDNA library. Bioinformatics analysis showed that BmRas1 contained an H-N-K-Ras-like domain belonging to Ras subfamily. Amino acid sequences of some members of the Ras family from many species were aligned. BmRas1 were highly homologous with ras protein of *Aedes aegypti* (EAT35784),* Anopheles gambiae *(XP_307965),  *Apis mellifera* (XP_394288), and *Drosophila melanogaster* (NP476699). The lysine-rich C-termini (last 24-25 amino acids), termed “the hypervariable region” (HVR), was highly varied, which was divided into the lipid anchor and the preceding linker domain. This domain was responsible for the membrane anchoring and intracellular trafficking of Ras protein, which interacted electrostatically with negatively charged phospholipids in the internal membrane leaflet [34]. The CAAX motif of BmRas1, CTLF, was different with all aligned sequences of other species. *Bombyx mori* Ras proteins were neither farnesylated nor palmitoylated but were geranylgeranylated [33].

BmRas1 gene was cloned into pET-28a (+) vector and expressed in E.coli cells. Ras protein with low intrinsic GTPase activity can bind to GTP to hydrolysis. The GTPase activity of purified recombinant BmRas1 protein was tested by FeSO_4_-(NH_4_)_2_MoO_4_ assay. The result showed that we successfully expressed the recombinant BmRas1 proteins which possessed an intrinsic GTP hydrolysis activity.

The expression of BmRas1 was detected during different developmental stages in *Bombyx mori*. BmRas1 protein was expressed in high level in egg, pupae, and moths, but in low expression in fifth instar larvae. In order to learn more about the distribution of BmRas1 protein, the expression levels of BmRas1 in various tissues of the fifth instar larvae were analyzed. BmRas1 proteins were expressed in tissues including malpighian tubule, head, seminal glands, ovary, and silk gland. The biological functions of BmRas1 protein in different tissues distribution need to be explored in further research. Subcellular localization of the protein can provide valuable clues about its function. The subcellular localization of the BmRas1 was examined by laser confocal microscopy. The result suggested that BmRas1 mainly localized on cellular membrane, partly in cytoplasm of BmN cells. The difference from Ogura's result that BmRas1 proteins were specifically localized on the cell membrane may be related to difference in cells used. Furthermore, Sf-9 cells were used in Ogura's experiment, while BmN cells were used in this research. Sf-9 Cells are derived from Spodoptera frugiperda, whereas BmN cells are derived from *Bombyx mori*. The basic research of the silkworm BmRas1 protein in tissue localization and biological functions will provide an important basis for further study of the role of this protein in silkworm.
